# Spatial specificity of metabolism regulation of abscisic acid-imposed seed germination inhibition in Korean pine (Pinus koraiensis sieb et zucc)

**DOI:** 10.3389/fpls.2024.1417632

**Published:** 2024-06-20

**Authors:** Yuan Song, Xinghuan Li, Mingyi Zhang, Chao Xiong

**Affiliations:** ^1^ College of Eco-Environmental Engineering, Guizhou Minzu University, Guiyang, China; ^2^ The Karst Environmental Geological Hazard Prevention Laboratory of Guizhou Minzu University, Guiyang, China; ^3^ Department of Health Management, Guiyang Institute of Information Science and Technology, Guiyang, China

**Keywords:** seed germination, abscisic acid, metabolism, radicle, hypocotyl, cotyledon

## Abstract

**Introduction:**

Abscisic acid (ABA) can negatively regulate seed germination, but the mechanisms of ABA-mediated metabolism modulation are not well understood. Moreover, it remains unclear whether metabolic pathways vary with the different tissue parts of the embryo, such as the radicle, hypocotyl and cotyledon.

**Methods:**

In this report, we performed the first comprehensive metabolome analysis of the radicle and hypocotyl + cotyledon in Pinus koraiensis seeds in response to ABA treatment during germination.

**Results and discussion:**

Metabolome profiling showed that following ABA treatment, 67 significantly differentially accumulated metabolites in the embryo were closely associated with pyrimidine metabolism, phenylalanine metabolism, cysteine and methionine metabolism, galactose metabolism, terpenoid backbone biosynthesis, and glutathione metabolism. Meanwhile, 62 metabolites in the hypocotyl + cotyledon were primarily involved in glycerophospholipid metabolism and glycolysis/gluconeogenesis. We can conclude that ABA may inhibit Korean pine seed germination primarily by disrupting the biosynthesis of certain plant hormones mediated by cysteine and methionine metabolism and terpenoid backbone biosynthesis, as well as reducing the reactive oxygen species scavenging ability regulated by glutathione metabolism and shikimate pathway in radicle. ABA may strongly disrupt the structure and function of cellular membranes due to alterations in glycerophospholipid metabolism, and weaken glycolysis/gluconeogenesis in the hypocotyl + cotyledon, both of which are major contributors to ABA-mediated inhibition of seed germination. These results highlight that the spatial modulation of metabolic pathways in Pinus koraiensis seeds underlies the germination response to ABA.

## 1 Introduction

Seed germination introduces seed plant into natural ecosystems, marking the beginning of their life cycle (Hao et al., 2022). This process is highly susceptible to diseases and environmental stresses (Liu et al., 2015). Thus, most plants have evolved seed dormancy mechanisms to prevent seedling emergence under unsuitable environmental conditions and initiate the new life cycle after sensing optimal environmental cues (Bentsink and Koornneef, 2008; Nonogaki et al., 2018). Based on the different times when seed dormancy occurs, seed dormancy can be classified into primary dormancy (developed during seed maturation) and secondary dormancy (induced after seed shedding) (Bewley et al., 2013). It is commonly known that ABA (abscisic acid) induces primary dormancy during seed maturation and inhibits germination during imbibition (Finkelstein et al., 2008). Therefore, it is of great theoretical and practical importance to reveal the mechanism by which ABA inhibits germination.

Although the time required for seed germination is relatively short compared with other stages in the plant life cycle, the underlying mechanism is complicated. Seed germination is a process during which the heterotrophic state is gradually transformed to an autotrophic state (Nonogaki et al., 2010; Shu et al., 2016; Carrera-Castaño et al., 2020), requiring a large amount of energy and carbon skeletons (Nonogaki et al., 2010; Weitbrecht et al., 2011). Metabolites can provide raw materials for diverse metabolic and cellular events, including DNA damage and repair, phytohormone metabolism and signal transduction, nutrient and energy metabolism, as well as cell wall remodeling and modification (Guo and Gan, 2005; Guo et al., 2021). Germinating seeds are in a highly active metabolic state. Undoubtedly, metabolites play key roles in seed germination. ABA can strongly affect the process of seed germination by modulating ABA-related metabolites. Thus, knowing the key metabolites as well as their pathways involved in modulation of ABA is important for understanding the mechanism of seed germination inhibited by this important exogenous phytohormone.

Transcriptomic, proteomic and metabolomic analyses are effective methods for examining simultaneous metabolic changes occurring during complex germination processes with high throughput and efficiency (Liu et al., 2015; Gorzolka et al., 2016; Kazmi et al., 2017; Silva et al., 2017; Chen et al., 2019). Along with the rapid development of systems biology and considerable progress in high-throughput sequencing, integrated omics have been increasingly used in studies on the mechanisms of seed development, dormancy and germination. In previous studies, transcriptomics, proteomics and/or metabolomics analysis of seed germination in the presence of ABA have been extensively conducted. Most studies have focused on herbaceous species, such as Arabidopsis (Garciarrubio et al., 1997; Pritchard et al., 2002; Wu et al., 2020a, b; Xue et al., 2021; Chen et al., 2022; Gong et al., 2022; Li et al., 2023), rice (*Oryza sativa*) (Kim et al., 2008; Liu et al., 2015; Wang et al., 2020), wheat (*Triticum aestivum*) (Yu et al., 2016), barley (*Hordeum vulgare*) (Huang et al., 2016), *Medicago truncatula* (Gimeno-Gilles et al., 2009), *Astragalus membranaceus* (Yang et al., 2018) and *Panax notoginseng* (Wang et al., 2023). By contrast, information on trees, such as pear (*Pyrus pyrifolia* (Burm.f.) Nakai) (Qi et al., 2022), *Magnolia sieboldii* (Lu et al., 2023), European beech *(Fagus sylvatica)* (Pawłowski, 2007), and Norway maple (*Acer platanoides*) (Pawłowski, 2009) remains limited. Based on these studies, it is concluded that ABA plays at least three roles in inhibiting seed germination. First, ABA prevents the mobilization of storage reserves, such as lipid, starch, and protein (Garciarrubio et al., 1997; Yu et al., 2016; Yang et al., 2018), and inhibits the activities of enzymes involved in carbohydrate and energy metabolic pathways, including glycolysis/gluconeogenesis, citric acid cycle, glyoxylate cycle, and oxidative phosphorylation) (Garciarrubio et al., 1997; Qi et al., 2022; Lu et al., 2023), thus resulting in a reduction in energy and nutrient supplies to the embryo (Pawłowski, 2009; Liu et al., 2015; Wang et al., 2023). Second, ABA promotes the biosynthesis of JA (jasmonic acid) and IAA (indoleacetic acid), while it downregulates GA (gibberellin) synthesis (Shi et al., 2019) and GA signal transduction (Wang et al., 2023). Furthermore, it blocks the biosynthesis pathway of ethylene precursors (Pawłowski, 2009; Liu et al., 2015). Third, ABA not only induces the accumulation of ROS (reactive oxygen species) but also decreases the ROS scavenging ability (Wu et al., 2020b; Gong et al., 2022; Li et al., 2023). In addition to three aspects described above explaining ABA-imposed germination inhibition, ABA is known to affect cell division (Liu et al., 2015), interfere with the purine metabolism pathway (Cornelius et al., 2011; Qi et al., 2022), inhibit amino acid synthesis (Wang et al., 2023), and interfere with cell wall loosening and expansion (Gimeno-Gilles et al., 2009). These conclusions have laid a solid foundation for deeper insights into the mechanisms by which ABA inhibits woody-plant seed germination. Furthermore, few studies reveal that there is a tissue-specific response of metabolic pathways to ABA sensitivity between the embryo and endosperm (Manz et al., 2005; Penfield et al., 2006; Yu et al., 2016). For example, the ABA-responsive metabolic pathways in the embryo included DNA synthesis, carbohydrate metabolism, hormone metabolism, and protein degradation, while in the endosperm the metabolism related to storage reserve mobilization, transport, biotic and abiotic stresses, hormone metabolism, cell wall metabolism, signaling, and development were more predominant (Yu et al., 2016). Compared with embryonic ABA, endospermic ABA seems to play minimal role in inhibiting seed germination (Izydorczyk et al., 2018). A recent and detailed study showed that ABA-mediated germination inhibition is due to the limit of local glucose supply in the hypocotyl region of embryo (Xue et al., 2021). Therefore, to gain an insight into the inhibition mechanism of ABA on germination, it is necessary to study the metabolic response to ABA in different embryo tissues, such as the radicle, hypocotyl and cotyledons.

Korean pine (*Pinus koraiensis*) is an ecologically and economically significant coniferous species that is widely distributed in China, Russia, Korea, and Japan (Wang, 1995). Korean pine could be used for the extraction of rosin, turpentine and pine needle oil (Wu et al., 1996). Moreover, Korean pine seeds possess high nutritional and certain medicinal value due to their high levels of vitamins, such as vitamin A and E (Shen, 2002; Xu et al., 2014), and unsaturated fatty acids, including linoleic acid, linolenic acid and pinolenic acid (Shen, 2002; Bao et al., 2020). Previous research has indicated that excessive logging of woody plants and the low germination percentage induced by seed dormancy have resulted in low regeneration of Korean pine individuals in the natural ecosystem (Zhu et al., 2007). Morphophysiological dormancy (physiological inhibiting factors in the undifferentiated or underdeveloped embryo) is observed in fresh Korean pine seeds when they disperse in autumn (Song et al., 2023). Moist chilling, where imbibed seeds were mixed with moist sand and then stored under low temperature conditions for approximately six months, is widely applied to reduce seed dormancy and promote seed germination of Korean pine (Wang, 1995). Although the inhibition of Korean pine seed germination and the regulation of seed dormancy status by ABA has been widely studied (Lai et al., 1989; Song et al., 2018), the underlying mechanisms remain incompletely understood. The objective of this study was to employ non-targeted metabolomics to identify the metabolic regulation mechanism underlying the inhibition of ABA on the germination of *Pinus koraiensis* seeds. We thus compared the metabolome of Korean pine seeds germinating at 25/15°C with that of Korean pine seeds whose germination was inhibited by treatment with exogenous ABA. We then identified the differentially accumulated compounds between untreated control and ABA-treated radicle and hypocotyl + cotyledons samples. The metabolic pathways associated with the ABA response were screened in radicle and hypocotyl + cotyledons, respectively. This information is useful for elucidating the spatial specificity of metabolomic regulation mechanisms of inhibiting seed germination by ABA.

## 2 Materials and methods

### 2.1 Plant material

In late-September (early-autumn) 2020, Korean pine seeds were collected from at least 50 Korean pine trees at the ripening stage in a mixed broad-leaved Korean pine forest (MBKPF) in Liangshui National Nature Reserve (47°6′49”N—47°16′10″N, 128°47′8″E—128°57′19”E) in northeastern China. Regional climate is characterized by temperate continental monsoon. The water content of the seed was less than approximately 10%, whereas its viability exceeded 90%. Seeds were then stored at -20°C for about one month to maintain their primary physiological dormancy status.

### 2.2 Seed primary physiological dormancy breaking treatment

We previously showed that under natural conditions, the primary physiological dormancy of fresh Korean pine seeds falling off in early-autumn was progressively released during subsequent autumn and winter (Song et al., 2023). Based on this observation, in early October, Korean pine seeds were taken out of -20°C storage conditions and then buried between litterfall and soil in MBKPF for about eight months, aiming to release their primary physiological dormancy in the presence of moist cold conditions in autumn and winter.

The specific procedures were as follows. First, three sites, approximately 50 m apart from each other, were selected as seed burial points in MBPKF. Second, 5000 seeds were mixed with soil from MBKPF. The mixture of seeds and soil was then placed in a nylon bags (50 cm in length × 50 cm in width, with 3 mm aperture). Third, this nylon bag containing seeds and soil was then placed in a metal container to prevent predation from animals. One nylon bag was buried in each seed burial site, serving as a replicate. A total of three replicates was set up in the present study, with 5000 seeds in each replicate.

### 2.3 Determination of seed primary physiological dormancy release

After eight months, in late May in the second year of seed burial, seeds were retrieved from each burial site and then used for germination tests to evaluate if these seeds had already been released from primary physiological dormancy. Seed dormancy release can be characterized by a gradual widening of the temperature range that permits germination to occur (Baskin and Baskin, 2014). Thus, four fluctuating temperature regimes mimicking the approximate mean maximum and minimum temperatures for each season in the Liangshui region were used for germination tests. 10/5°C, 20/10°C, 25/15°C and 30/20°C represent mid-spring/late-autumn, late-spring/early-autumn, early-summer and mid-summer temperature, respectively. Three replicates of 20 seeds each were incubated on five layers of filter paper moistened with 8 mL of distilled water in closed 100-mm diameter Petri dishes at four fluctuating temperature regimes for about eight weeks. For each fluctuating temperature regime, the duration at the maximum and minimum temperature was 14 and 10 h, respectively. Correspondingly, during the 14 h light period (200 μmol m^-2^ s^-1^), the temperature was set to the maximum, while during the 10 h dark period, the temperature was set to the minimum. Seeds were checked every two days, and a radicle length of 2 mm was used as the criterion for completion of germination. These germinated seeds were counted and removed from the petri dish. At the same time, distilled water was added as required to keep seeds moisture. Germination was considered complete when seeds did not germinate for three consecutive days. After the germination test was terminated, the ungerminated seeds were dissected to determine the viability of the embryo. Seeds with viable embryos were used to compute the seed germination percentage. Seeds containing white and firm embryos are considered alive. If embryos are soft and brown, these seeds are considered dead.

### 2.4 ABA treatment of seeds with released primary physiological dormancy

Seeds that had released their primary physiological dormancy were collected from seed burial sites in late-May in the second year of seed burial. These seeds were then treated with either distilled water (control) or 1 mM ABA (ABA-treatment). Five replicates, with 200 seeds per replicate, were set up for both the control and the ABA-treatment groups, respectively. Every 200 seeds were placed on a sterile medical cotton pad wetted with distilled water, in sealable, clear, plastic container (23 cm long × 20 cm wide × 5 cm high). The containers were then incubated at the optimal temperature of 25/15°C (day/night) under a 14/10-hour light/dark cycle, with a 14-hour photoperiod and approximately 200 μmol of photons m^-2^ s^-1^ for 2 weeks. After 2 weeks of incubation, the embryo of the control and ABA-treatment seeds were dissected into radicle and hypocotyl + cotyledon. The radicle and hypocotyl + cotyledon samples were frozen in liquid nitrogen and stored at -80°C until use.

### 2.5 Sample preparation for metabolomic analysis

After the samples were slowly thawed at 4°C, pre-cooled acetonitrile/methanol/aqueous solution (2:2:1, v/v/v) were added to the samples (Pi et al., 2018). The mixture of samples and extraction solution was first vortexed, and then subjected to cryogenic sonication treatment for 30 min (twice) (Pi et al., 2018). Then they were left to stand at -20°C for 60 min and subsequently centrifuged at 14,000 g at 4°C for 20 min to obtain the supernatant (Pi et al., 2018). The supernatant was lyophilized and stored at -80°C until further analysis. When liquid chromatography-mass spectrometry analysis was conducted, the freeze-dried powder was first redissolved in 100 μL of a 1:1 (v/v) acetonitrile:water solution, then vortexed, and subsequently centrifuged at 14000 g for 15 min at 4°C (Gu et al., 2018). After that the supernatant was taken for analysis.

### 2.6 Liquid chromatography-mass spectrometry analysis

LC-MS analysis was performed using an Agilent 1290 Infinity ultra-high performance liquid chromatography (Agilent Technologies, Santa Clara, CA, United States) coupled to a time-of-flight mass spectrometer (Triple TOF 6600, AB SCIEX, Framingham, MA, United States) (Wu et al., 2020a). The sample analytes were separated on a hydrophilic interaction liquid chromatography (HILIC) column (ACQUITY UPLC BEH Amide 2.1 mm × 100 mm column, internal diameter 1.7 μm, Waters, Ireland) at a constant flow rate of 0.5 mL·min^-1^ (Wu et al., 2020a). The column temperature was maintained at 25°C, and the sample injection volume was 2 μL (Wu et al., 2020a). The mobile phase comprised aqueous ammonium acetate (25 mM)/ammonia (25 mM) (A) and acetonitrile (B) (Wu et al., 2020a). The gradient elution program optimized for separation was as follows: 0–0.5 min, 95% B; 0.5–7 min, 95–65% B; 7–8 min, 65–40% B; 8–9 min, 40% B; 9–9.1 min, 40–95% B; 9.1–12 min, 95% B (Wu et al., 2020a). The samples were kept in a 4°C autosampler tray during the entire analysis process (Wu et al., 2020a).

Following HILIC chromatographic separation, the electrospray ionization (ESI) source operation parameters were set as follows: ion source gas1 (Gas1) and ion source gas2 (Gas2) were both set to 60, curtain gas (CUR) to 30, and the source temperature to 600°C. The IonSapary Voltage Floating (ISVF) was ± 5500 V for both positive and negative ion mode. The TOF MS scan m/z range was set from 60 to 1000 Da, while the product ion scan m/z range was from 25 to 1000 Da. The TOF MS scan accumulation time was 0.20 s per spectrum, and for product ion scan accumulation time, it was 0.05 s per spectrum (Fan et al., 2018). MS/MS data was acquired using information-dependent acquisition (IDA) with high sensitivity mode selected (Fan et al., 2018). The parameters were set as follows: the collision energy (CE) was fixed at 35 ± 15 eV, the declustering potential (DP) was ± 60 V (positive and negative ion mode), isotopes within 4 Da were excluded and 10 candidate ions were monitored per cycle (Fan et al., 2018).

### 2.7 Data processing

The raw data (wiff.scan files) were converted to MzXML files by ProteoWizard MSConvert. Peak alignment, retention time correction and peak area extraction were conducted with the XCMS program. The structure of metabolite was identified by matching the mass-to-charge ratio, retention time, molecular mass (molecular mass error within < 25 ppm), secondary fragmentation spectra, and collision energy of the sample metabolite with the metabolite in in-house database (Shanghai Applied Protein Technology) (Gu et al., 2018).

### 2.8 Statistical analysis

Partial least squares-discriminant analysis (PLS-DA) was carried out on the control and ABA-treatment samples to reveal the difference in metabolite profiles among the radicle of control embryos (abbreviated R) and ABA-treated embryos (abbreviated RA), the hypocotyl + cotyledon of control embryos (abbreviated HC) and ABA-treated embryos (abbreviated HCA). The metabolite data were log transformed (generalized logarithm transformation) and auto scaled (mean-centered and divided by the standard deviation of each variable) for normalization before performing PLS-DA analysis. PLS-DA was also conducted to determine the differentially expressed metabolites between R and RA, as well as between HC and HCA. The metabolite data were pareto scaled (mean-centered and divided by the square root of the standard deviation of each variable) for normalization before performing PLS-DA. The key differentially expressed metabolites were selected based on two criteria: variable importance in the projection (VIP) values greater than 1 and *P*-values from Student’s t-test less than 0.05. Fold change in the relative contents of these differentially expressed metabolites was calculated between R and RA, and also between HC and HCA. Subsequently, those metabolites with VIP greater than 1 and *P*-value less than 0.05 were also subjected to metabolic pathway analysis to determine the metabolic pathways in which they are enriched. PLS-DA, the calculation of fold change and the metabolic pathways analysis were all conducted using the MetaboAnalyst 5.0. For the metabolic pathways analysis, the corresponding parameters were set according to the methods described by Song and Zhu (2019). The most relevant metabolic pathways were identified by applying the criteria of a *P*-value less than 0.05 and an impact-value threshold greater than 0.1 (Wang et al., 2012).

## 3 Result

### 3.1 Germination of seeds with released primary physiological dormancy and seeds treated with ABA

A total of 66.67 ± 10.41%, 83.33 ± 7.64% and 56.67 ± 15.28% of seeds germinated at 20/10°C, 25/15°C and 30/20°C, respectively. However, only about 15.00% of seeds germinated at 10/5°C. Notably, none of the seeds treated with 1 mM ABA germinated under any of these four temperature regimes.

### 3.2 Partial least squares-discriminant analysis of metabolites in the control and ABA-treated seeds

**Figure 1:**
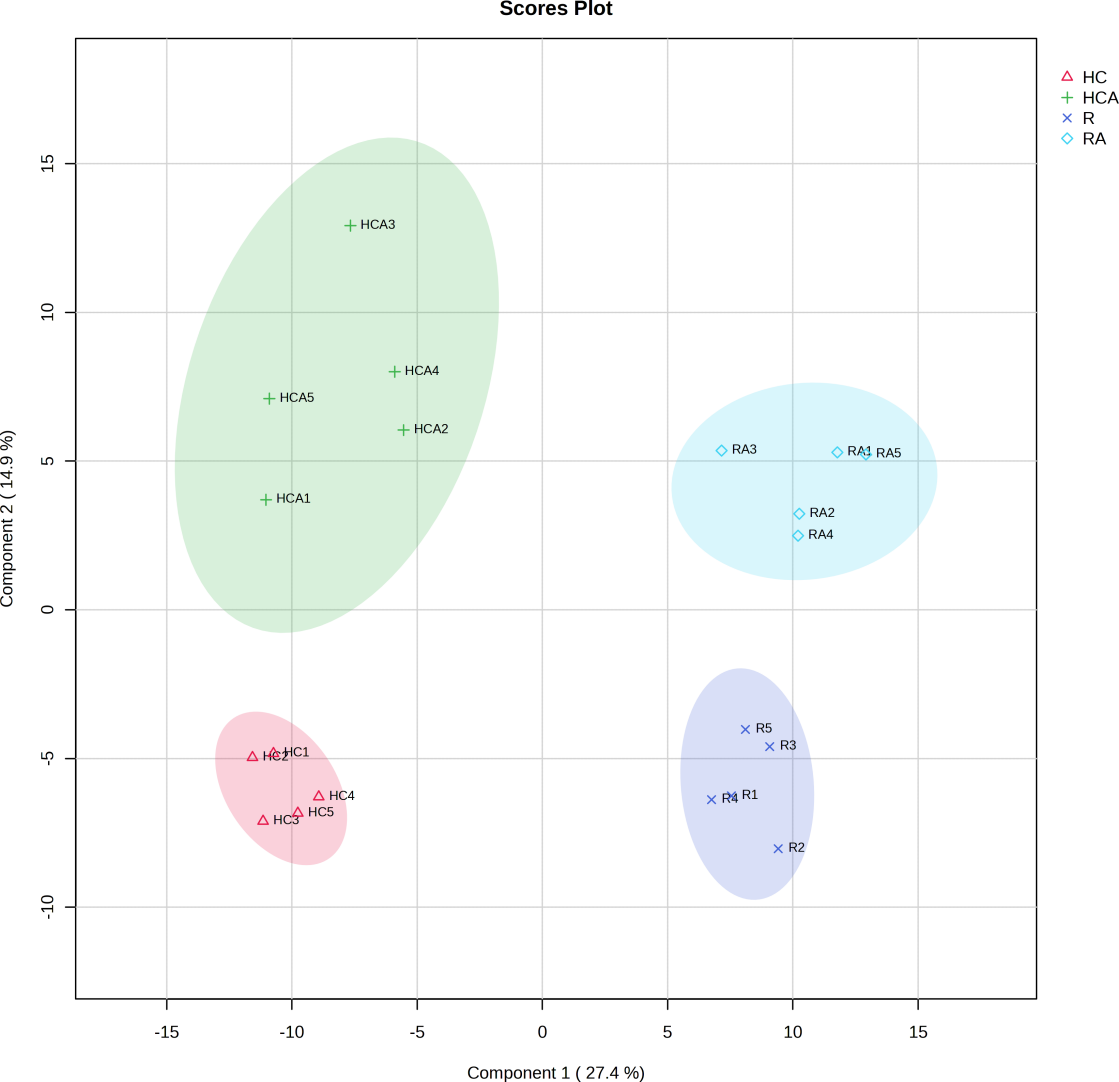
Principal least squares-discriminant analysis of measured metabolites in the radicle of control seeds (R), the radicle of ABA-treated seeds (RA), the hypocotyl + cotyledon of control seeds (HC), and the hypocotyl + cotyledon of ABA-treated seeds (HCA).

PLS-DA of the total metabolite data revealed differences in metabolites among samples. The PLS-DA of all the measured metabolites demonstrated that the five replicates of control and ABA-treated radicle (or hypocotyl + cotyledon) were clustered together separately (**Figure 1**), indicating similarity among replicates within each group. The first principal component (pca1) separated the radicle and hypocotyl + cotyledon groups, with a contribution value reaching 27.4%. The second principal component (pca2) had a contribution value of 14.9% (**Figure 1**). The clear distinction was observed on the second principal component between the control and ABA-treated radicle groups, as well as between the control and ABA-treated hypocotyl + cotyledon groups (**Figure 1**).

### 3.3 Fold changes of metabolites showing significant changes between the control and ABA-treated seeds

When seeds were germinated for 2 weeks at 25/15°C, which is the optimal temperature for Korean pine seed germination, a total of 67 metabolites were identified as having significantly altered relative contents in the ABA-treated radicle compared to the control radicle (**Table 1** and **Supplementary Table S1**). It was found that the relative amounts of 20 metabolites were significantly higher in the ABA-treated radicle compared to the control (**Table 1**). These metabolites that showed significant increases in relative levels were mainly classified into nucleosides, nucleotides, and analogues (nine in total), lipids and lipid-like molecules (three in total), and organic acids and derivatives (three in total). After ABA treatment, the ABA relative level in the radicle was approximately 10-fold higher. Additionally, 47 metabolites exhibited relatively lower contents in the ABA-treated radicle compared to the control radicle (**Table 1**). Specifically, the most numerous metabolites were organic acids and derivatives (14 in total), followed by lipids and lipid-like molecules (eight in total), and carbohydrates and carbohydrate conjugates (five in total), organoheterocyclic compounds (five in total) and benzenoids (three in total). Six organic acids and derivatives, including L-leucine, 5-L-glutamyl-L-alanine, gamma-glutamyl-L-methionine, L-valine, (R)-mevalonic acid 5-phosphate and 3-hydroxycapric acid, were observed to show at least a 1.5-fold decrease. S-methyl-5’-thioadenosine, a member of the nucleosides, nucleotides, and their analogues, exhibited the most significant reduction, with a 3.20-fold drop.

**Table 1:** Information on 67 metabolites with VIP > 1 and *P* < 0.05 is presented for the comparison between the radicle of control seeds and the radicle of ABA-treated seeds.

Number	Metabolites	Fold change	VIP	*P* value
1	3-Dehydroshikimic acid	0.2863	1.7071	0.0037
2	9R,10S-Epoxy-17R-hydroxy-prosta-5Z,13E-dien-1-ol methyl ester	0.3028	1.7817	0.0016
3	S-Methyl-5’-thioadenosine	0.3125	2.1092	0.0015
4	3-Hydroxycapric acid	0.3415	1.5314	0.0153
5	Uridine 5’-monophosphate	0.3451	1.7567	0.0021
6	1,4-Dihydroxybenzene	0.3573	1.6390	0.0069
7	Phenylpyruvate	0.3845	1.7927	0.0014
8	(R)-mevalonic acid 5-Phosphate	0.3919	1.3185	0.0493
9	N-Acetylmannosamine	0.4766	1.6948	0.0277
10	Mevalonic acid	0.4914	1.5243	0.0161
11	Shikimate	0.5069	1.9491	0.0001
12	Pantothenol	0.5128	1.7713	0.0189
13	Uracil	0.5211	1.9965	0.0044
14	L-Gulonolactone	0.5339	1.7214	0.0032
15	Eicosatrienoic Acid	0.5549	1.5764	0.0112
16	L-Valine	0.5703	1.6032	0.0413
17	gamma-Glutamyl-L-Methionine	0.5751	1.1702	0.0007
18	5-L-Glutamyl-L-alanine	0.5760	1.4796	0.0212
19	Salicylic acid	0.5926	1.8464	0.0006
20	Lithocholic acid	0.5936	1.344	0.0438
21	L-iditol	0.6266	1.6772	0.0049
22	Urocanic acid	0.6274	2.1352	0.0011
23	alpha-D-Glucose	0.6300	1.3678	0.0390
24	3-(3-Hydroxyphenyl) propanoic acid	0.6345	1.5964	0.0097
25	Dihomo-gamma-Linolenic Acid	0.6362	1.6525	0.0061
26	L-Arabinono-1,4-lactone	0.6382	1.7005	0.0039
27	Pyruvaldehyde	0.6392	1.7927	0.0336
28	L-Leucine	0.6585	2.1755	0.0007
29	Amygdalin	0.6747	1.7017	0.0039
30	3,3’,4’5-Tetrahydroxystilbene	0.6952	1.6264	0.0076
31	L-Alanine	0.6960	1.7341	0.0229
32	N-Carboxyethyl-g-aminobutyric acid	0.7026	1.9266	0.0074
33	Allantoin	0.7048	1.7808	0.0016
34	Heptadecanoic acid	0.7225	1.6735	0.0051
35	L-Isoleucine	0.7238	1.6118	0.0086
36	Tyramine	0.7417	2.1082	0.0015
37	trans-Zeatin	0.7830	1.8129	0.0151
38	Azelaic acid	0.8003	1.3748	0.0377
39	DL-2-Aminoadipic acid	0.8135	1.5983	0.0095
40	(E)-4-Hexen-1-ol	0.8151	1.9339	0.0070
41	Palmitic acid	0.8284	1.3497	0.0426
42	3-Phosphoserine	0.8359	1.4279	0.0286
43	Glutaric acid	0.8488	1.5610	0.0125
44	L-Pyroglutamic acid	0.8529	1.6212	0.0383
45	Diethanolamine	0.8679	1.6744	0.0304
46	N-Acetyl-D-glucosamine	0.8714	1.6308	0.0277
47	L-Ribulose	0.8870	1.4697	0.0225
48	Trigonelline	1.2620	1.6774	0.0300
49	Guanosine	1.2667	1.7413	0.0221
50	Glutathione disulfide	1.3017	1.6947	0.0277
51	Pro-Ser	1.3400	1.5860	0.0443
52	Cytidine	1.3511	1.6187	0.0387
53	L-Methionine	1.3535	1.8756	0.0104
54	2’-Deoxy-D-ribose	1.5224	1.5153	0.0170
55	Adenosine monophosphate	1.6606	1.4882	0.0202
56	Nicotinamide adenine dinucleotide (NAD)	1.7105	1.7821	0.0179
57	UDP-N-acetylglucosamine	1.7543	1.8863	0.0003
58	beta-Estradiol	1.8219	1.5694	0.0473
59	3’-O-methyladenosine	1.8480	1.9488	0.0063
60	Arg-Thr	1.8692	1.6627	0.0320
61	Cytidine 5’-diphosphocholine (CDP-choline)	1.9492	1.9156	0.0080
62	L-Asparagine	2.0075	1.7084	0.0259
63	Dodecanoic acid	2.1723	1.8293	0.0008
64	UDP-D-Galactose	2.3623	2.0315	0.0033
65	Ethyl glucuronide	2.7903	1.7420	0.0025
66	Uridine 5’-diphosphate	2.9389	2.2003	0.0021
67	(+)-Abscisic acid	10.1308	1.9809	0.0000

Fold changes (FC) were calculated as the relative levels in the ABA treatment group compared to those in the control group. A fold change greater than 1 indicates a relatively higher concentration in the ABA treatment group, whereas a fold change less than 1 indicates a concentration lower than that in the control group. VIP stands for variable influence on projection. “Arg-Thr (Pro-Ser)” refers to the formation of a dipeptide when arginine and threonine (or proline and serine) residues are linked together through a peptide bond.

A total of 62 ABA-responsive metabolites in the hypocotyl + cotyledon were identified when comparing the germination of Korean pine seeds treated with ABA to germination in distilled water, both at 25/15°C, after 2 weeks (**Table 2** and **Supplementary Table S2**). Our results revealed that seven metabolites were accumulated and 55 metabolites decreased in the hypocotyl + cotyledon of ABA-treated seeds compared to the control group. The relative content of ABA in the hypocotyl + cotyledon was increased by at least 10-fold in ABA-treated seeds, in comparison to the control group. Furthermore, there was also a 1- to 3-fold increase in the relative levels of methylmalonic acid, dodecanoic acid, isobutyric acid, meso-tartaric acid, myristic acid, and (R)-mevalonic acid 5-phosphate following ABA treatment. Fifty-five metabolites with a significant decrease in relative levels were classified into lipids and lipid-like molecules (25), organic acids and derivatives (seven), carbohydrates and carbohydrate conjugates (six), nucleosides, nucleotides, and analogues (five), benzenoids and phenylpropanoids (two), and polyketides (two). Furthermore, the relative levels of approximately 12 lipids and lipid-like molecules, including cholesteryl sulfate, 15-Keto-prostaglandin E1, eicosapentaenoic acid, eicosadienoic acid, oleanolic acid, tetracosanoic acid, tricosanoic acid, behenic acid, eicosatrienoic acid, arachidic acid, 2-hydroxy-butanoic acid and heneicosanoic acid were reduced by more than 2-fold. Seven organic acids and derivatives mainly comprised three amino acids, namely gamma-glutamyl-L-methionine, L-isoleucine, and argininosuccinic acid, along with (S)-Lactate and phosphoenolpyruvate. Other carbohydrates and carbohydrate conjugates that were found to decrease significantly mainly comprised threonic acid, L-iditol, alpha-D-glucose, 2’-deoxy-D-ribose, 3,3’,4,5-tetrahydroxy-trans-stilbene, and L-ribulose, with decreases ranging from 1.5- to over 3.4-fold.

**Table 2:** Information on 62 metabolites with VIP > 1 and *P* < 0.05 is presented for the comparison between the hypocotyl + cotyledon of control seeds and the hypocotyl + cotyledon of ABA-treated seeds.

Number	Metabolites	Fold change	VIP	*P* value
1	(+)-Abscisic acid	10.2610	1.7538	0.0000
2	Methylmalonic acid	3.1681	1.5936	0.0011
3	Dodecanoic acid	2.2790	1.6136	0.0008
4	Isobutyric acid	1.9439	1.1709	0.0469
5	meso-Tartaric acid	1.4608	1.1667	0.0479
6	Myristic acid	1.2907	1.1842	0.0436
7	(R)-mevalonic acid 5-Phosphate	1.2421	1.1603	0.0495
8	Phosphoenolpyruvate	0.8303	1.2524	0.0295
9	Adenosine	0.7796	4.2639	0.0019
10	Diethanolamine	0.7629	1.5026	0.0055
11	(S)-Lactate	0.7415	1.4033	0.0099
12	Argininosuccinic acid	0.7324	1.5489	0.0447
13	Citramalic acid	0.7267	1.2145	0.0369
14	4-androsten-17beta-ol-3-one glucosiduronate	0.7059	1.3150	0.0196
15	Confertifoline	0.7032	1.3751	0.0125
16	Succinate	0.6961	1.3181	0.0382
17	Thymidine	0.6948	1.2587	0.0283
18	2’-O-methylguanosine	0.6903	1.4063	0.0096
19	Arachidonic Acid (peroxide free)	0.6867	1.3823	0.0118
20	L-Isoleucine	0.6779	1.2508	0.0298
21	L-Ribulose	0.6687	1.3413	0.0162
22	hydrocortisone acetate	0.6597	1.6910	0.0001
23	Ribothymidine	0.6587	1.4606	0.0058
24	3,3’,4,5-tetrahydroxy-trans-stilbene	0.6553	1.2623	0.0277
25	Amygdalin	0.6466	1.4217	0.0084
26	2’-Deoxy-D-ribose	0.6321	1.2290	0.0339
27	Phosphatidate	0.6262	1.4887	0.0043
28	Pyruvaldehyde	0.5943	1.3729	0.0127
29	Phosphatidylcholine thioetheramide	0.5915	12.5550	0.0417
30	Heptadecanoic acid	0.5845	1.3351	0.0169
31	3-(3-Hydroxyphenyl) propanoic acid	0.5795	1.3111	0.0201
32	alpha-D-Glucose	0.5785	1.6475	0.0004
33	3’-O-methylguanosine	0.5771	1.3891	0.0111
34	gamma-Glutamyl-L-Methionine	0.5578	1.5539	0.0020
35	Dihomo-gamma-Linolenic Acid	0.5553	1.3909	0.0110
36	Phosphatidylcholine	0.5541	1.6202	0.0462
37	Tyramine	0.5452	1.3603	0.0037
38	9R,10S-Epoxy-17R-hydroxy-prosta-5Z,13E-dien-1-ol methyl ester	0.5350	1.2725	0.0260
39	1-Palmitoyl lysophosphatidic acid	0.5309	1.2656	0.0271
40	Prostaglandin A1	0.5283	1.5098	0.0034
41	Salicylic acid	0.5219	1.4647	0.0056
42	1-Acyl-sn-glycero-3-phosphocholine	0.5217	2.9371	0.0164
43	Pristanic acid	0.5134	1.1925	0.0417
44	Heneicosanoic acid	0.5112	1.4430	0.0069
45	2’-Deoxyuridine	0.4809	1.2290	0.0014
46	2-hydroxy-butanoic acid	0.4722	1.5313	0.0026
47	D-Arabinonic acid, gamma-lactone	0.4552	1.5931	0.0011
48	L-iditol	0.4506	1.2844	0.0241
49	Arachidic acid	0.4503	1.2163	0.0365
50	Eicosatrienoic Acid	0.4322	1.3435	0.0159
51	Behenic acid	0.4258	1.2495	0.0300
52	Tricosanoic acid	0.4099	1.2765	0.0253
53	Tetracosanoic acid	0.3922	1.3181	0.0191
54	Oleanolic acid	0.3849	1.5112	0.0034
55	Eicosadienoic Acid	0.3713	1.5106	0.0034
56	Hesperetin	0.3388	1.4572	0.0060
57	Eicosapentaenoic acid	0.3279	1.5799	0.0013
58	15-Keto-prostaglandin E1	0.3230	1.5497	0.0021
59	1,4-Dihydroxybenzene	0.3055	1.3768	0.0123
60	Threonic acid	0.2937	1.5620	0.0018
61	3-Dehydroshikimic acid	0.2099	1.5104	0.0034
62	Cholesteryl sulfate	0.1709	1.4127	0.0091

Fold changes (FC) were calculated as the relative levels in the ABA treatment group compared to those in the control group. A fold change greater than 1 indicates a relatively higher concentration in the ABA treatment group, whereas a fold change less than 1 indicates a concentration lower than that in the control group. VIP stands for variable influence on projection.

### 3.4 Significantly enriched metabolic pathways in the radicle and hypocotyl + cotyledon between the control and ABA-treated seeds

**Figure 2:**
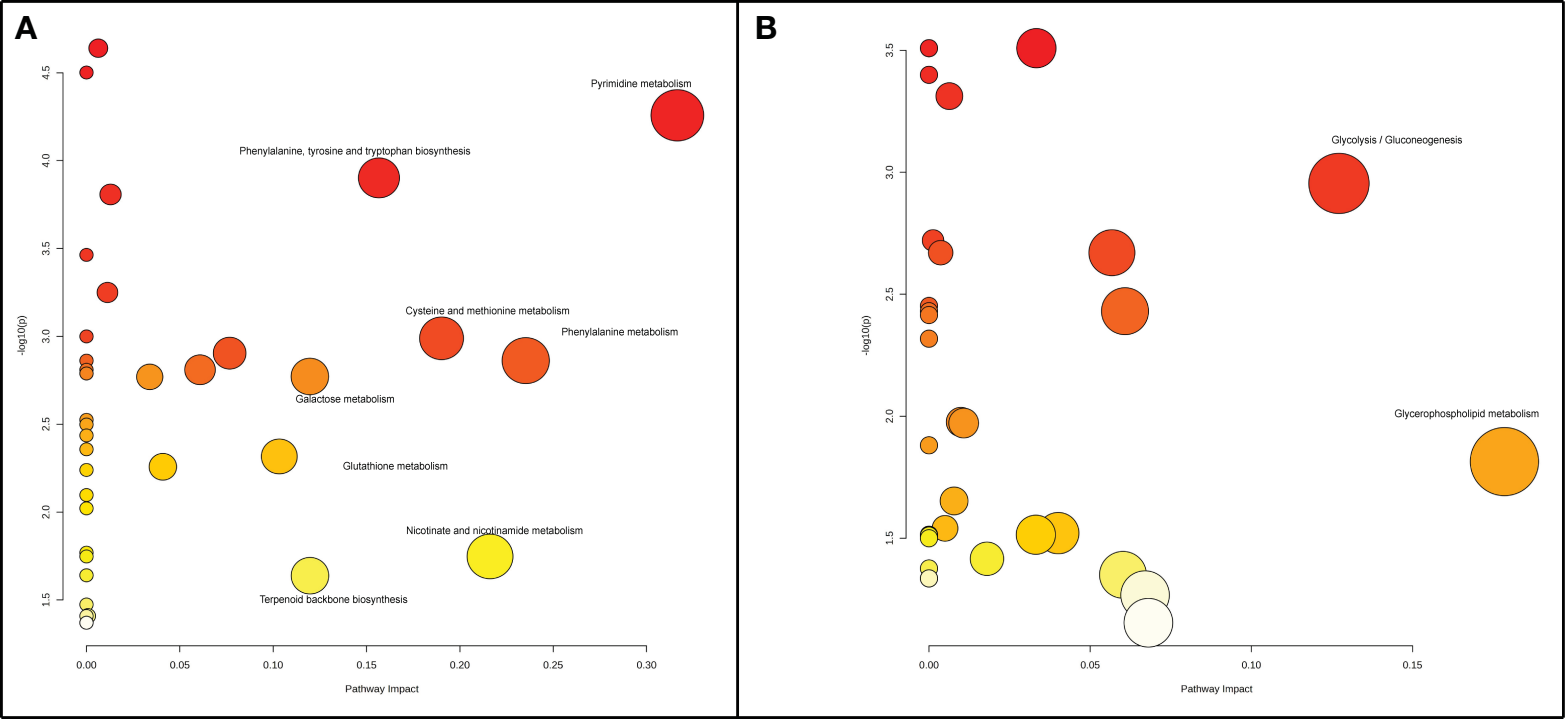
A Metabolome view depicts the altered metabolic pathways in **(A)** the radicle and **(B)** the hypocotyl + cotyledon of control seeds compared to ABA-treated seeds. Each node stands for a metabolic pathway. The color of the node signifies the *P*-value associated with each metabolic pathway, and the size (or radius) of the node represents the impact value of each metabolic pathway. Dark red, large circles located in the top right corner of the “metabolome view” indicate the primary altered pathways, in contrast to the yellow, small circles positioned on the left side of the graph represent the metabolic pathways that are less affected by ABA.

**Figure 3:**
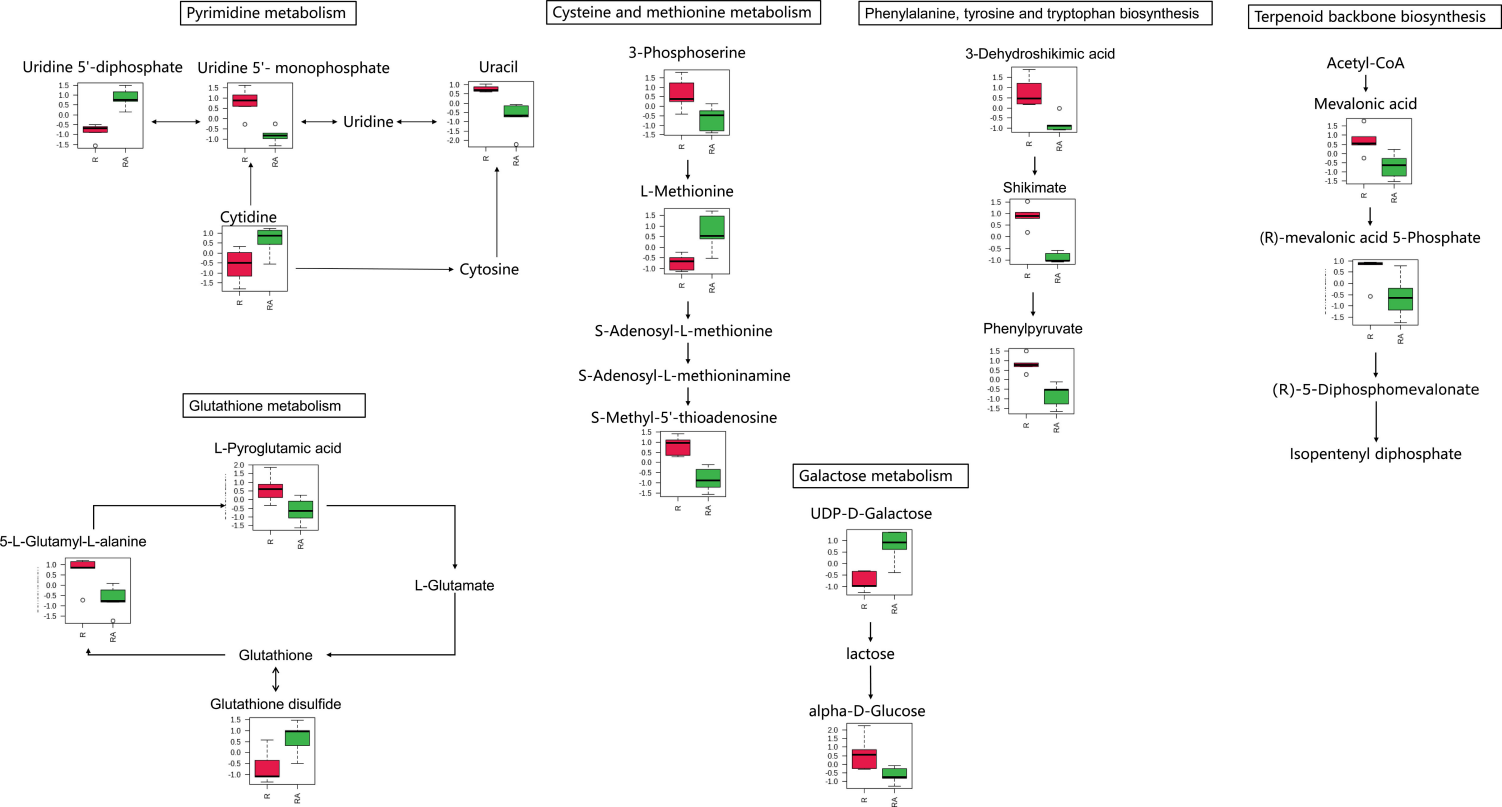
Metabolites were enriched in the eight altered pathway in the radicle of control seeds (R) compared to the radicle of ABA-treated seeds (RA). The relative contents of differentially expressed metabolites were first log transformed (using a generalized logarithm transformation) and then auto scaled (mean-centered and divided by the standard deviation of each variable) for normalization purposes. The normalized values are presented on the Y-axis. Since only nicotinamide adenine dinucleotide is enriched in nicotinate and nicotinamide metabolism, and phenylpyruvate is enriched in phenylalanine metabolism, these two pathways are not included.

**Figure 4:**
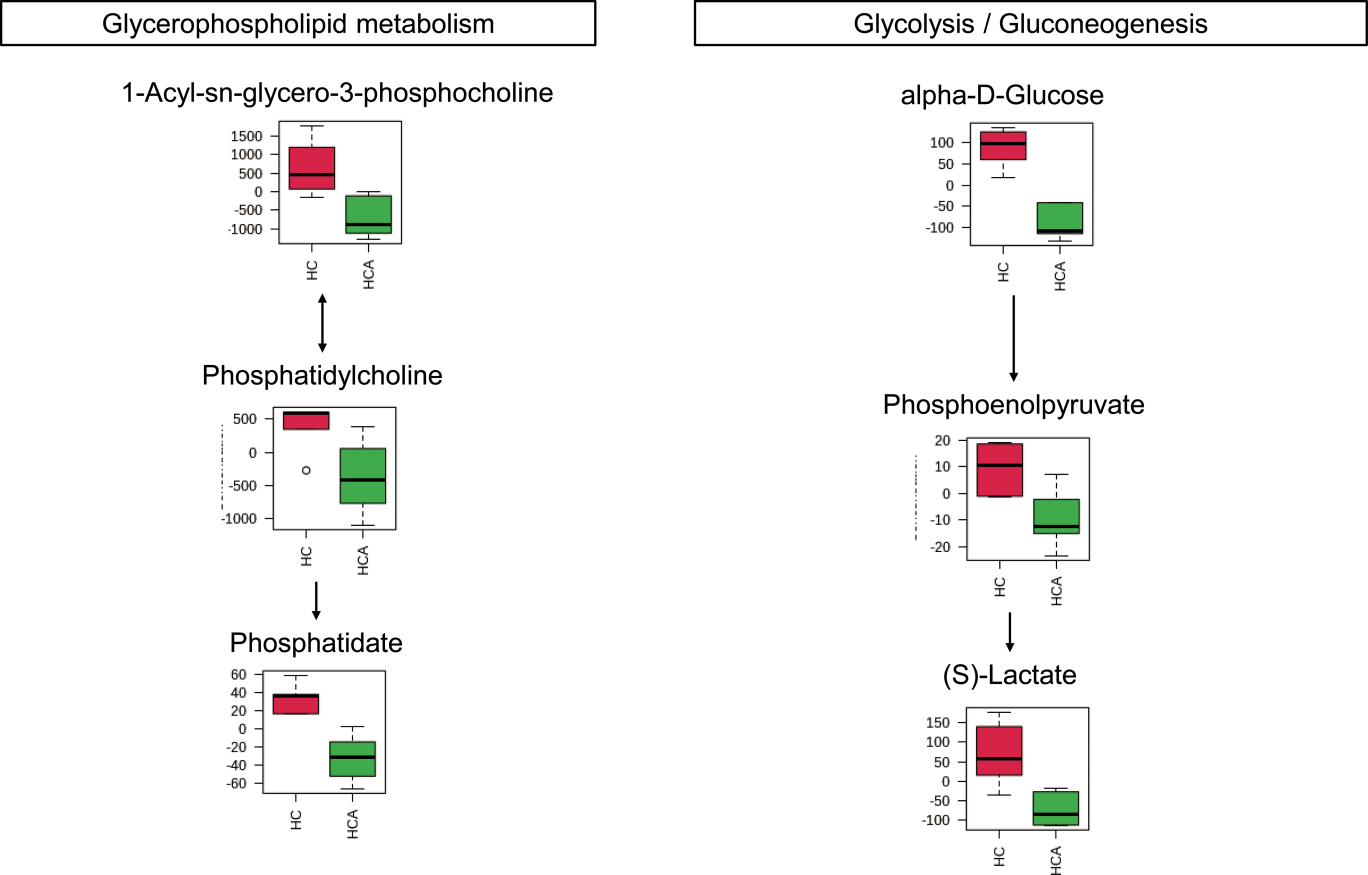
Metabolites were enriched in the two altered metabolic pathways in the hypocotyl + cotyledon of control seeds (HC) compared to the hypocotyl + cotyledon of ABA-treated seeds (HCA). The relative contents of differentially expressed metabolites were pareto scaled (mean-centered and divided by the square root of the standard deviation of each variable) for normalization purposes. The normalized values are presented on the Y-axis.

In the radicle, differentially expressed metabolites were enriched in eight metabolic pathways, including pyrimidine metabolism, phenylalanine metabolism, nicotinate and nicotinamide metabolism, cysteine and methionine metabolism, phenylalanine, tyrosine and tryptophan biosynthesis, terpenoid backbone biosynthesis, galactose metabolism, and glutathione metabolism (**Figures 2A**, **3**). Two metabolic pathways, namely glycerophospholipid metabolism and glycolysis/gluconeogenesis, were identified in the hypocotyl + cotyledon of ABA-treated seeds (**Figures 2B**, **4**).

## 4 Discussion

### 4.1 The change in metabolite profiles in the radicle during germination and their response to ABA treatment

**Figure 5:**
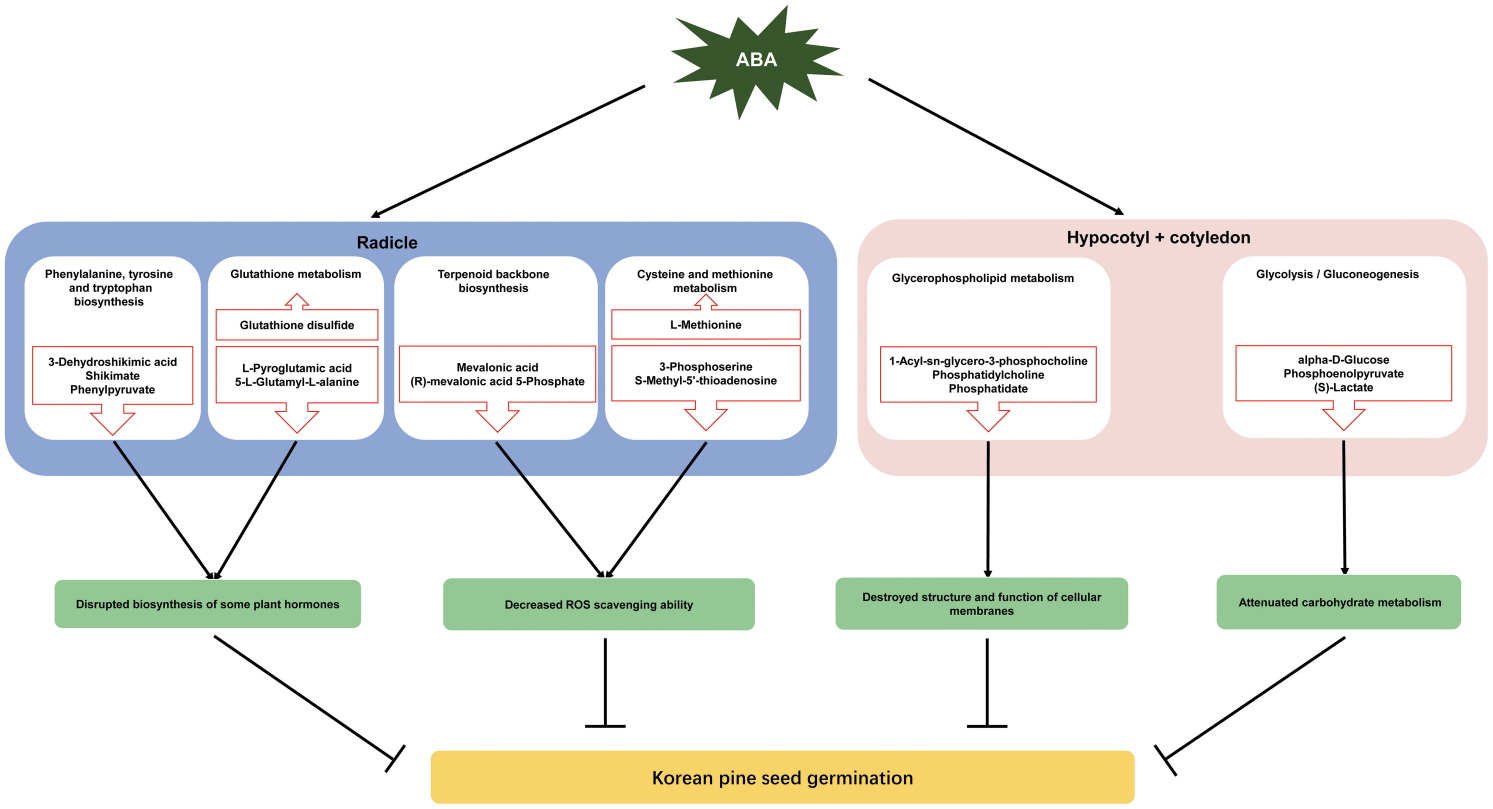
A Schematic diagram of metabolic regulation of ABA inhibition of Korean pine seed germination. The red upward arrows indicate upregulated metabolites, whereas the downward arrows represent the downregulated metabolites.

Approximately 70% of the metabolites with VIP > 1 and *P* < 0.05 were decreased under ABA treatment, indicating that ABA inhibited the metabolism of a large number of compounds in the radicle of Korean pine seeds. These metabolites were enriched in eight metabolic pathways, suggesting that these metabolic pathways may be affected by the ABA treatment. Furthermore, most of the metabolites involved in these pathways decreased under ABA treatment. These results further confirm that ABA may play an important role in the inhibition of Korean pine seed germination by disrupting certain pathways in the radicle, which mainly include phenylalanine, tyrosine and tryptophan biosynthesis, glutathione metabolism, terpenoid backbone biosynthesis, and cysteine and methionine metabolism (**Figure 5**).

It has been well documented that SAM (S-adenosylmethionine) functions as the universal methyl-group donor affecting the expression of genes related to seed dormancy breaking (Liu et al., 2015; Lu et al., 2018). In addition, SAM is also a precursor for the synthesis of various compounds, including the plant hormone ethylene, the vitamin biotin, the polyamines spermidine and spermine, as well as the iron chelator nicotianamine (Ravanel et al., 2008; Takahashi et al., 2011). Ethylene can antagonize ABA effects, thus promoting the breaking of seed dormancy and germination (Beaudoin et al., 2000; Lee et al., 2010b). The germination-promoting role of polyamines has been widely reported (Huang et al., 2017; Chen et al., 2019). S-adenosylmethionine synthetase catalyzes the biosynthesis of SAM from methionine and adenosine triphosphate (Gimeno-Gilles et al., 2009). During the biosynthesis of ethylene and polyamines, SAM produces S-methyl-5’-thioadenosine (MTA) as a byproduct. This MTA is then recycled to methionine via the Yang cycle (Miyazaki and Yang, 1987). Previous investigations have indirectly indicated that the metabolic process catalyzed by S-adenosylmethionine synthetase has a significant regulatory function when Arabidopsis seeds shift from a quiescent to a highly active state (Gallardo et al., 2003; Bouton et al., 2005).

In the present research, three metabolites related to the synthesis and breakdown of SAM have been identified in the metabolism of cysteine and methionine. The apparent accumulation of precursor of SAM biosynthesis, namely L-methionine, coupled with a significant reduction in the relative content of the product of SAM breakdown, MTA, in response to ABA, suggests that the SAM-related biosynthetic and catabolic pathways may be influenced by ABA. Moreover, the observed decline in 3-phosphoserine, the precursor of L-methionine, further supports the notion that the accumulation of L-methionine in ABA-treated radicles can be primarily attributed to the inhibition of SAM biosynthesis pathway. This observation led to the hypothesis that ABA inhibits Korean pine seed germination by preventing the biosynthesis of SAM, blocking methylation reactions in which SAM participates, or disrupting sulfur, ethylene, and polyamine biosynthesis. Our results resembled those of Pawłowski, who found that exogenously applied ABA down-regulates SAM synthetase and proposed that the negative regulation of ethylene synthesis caused by ABA-mediated processes is related to the inhibition of seed dormancy breaking in Norway maple (Pawłowski, 2009). A similar mechanism imposing an ABA requirement to inhibit germination was also reported in other plant species. For instance, a previous study on rice revealed that ABA treatment inhibited germination and reduced the abundance of proteins related to methionine metabolism (Liu et al., 2015). Mathur and Sachar (1991) reported that ABA significantly inhibited the increase in S-adenosylmethionine synthetase activity in dwarf pea (*Pisum sativum*). The position of the action of ABA is the epicotyls in the above research. It was also found that the proteins associated with s-adenosylmethionine synthetase accumulated in the hypocotyl during seed germination (Gimeno-Gilles et al., 2009). However, our metabolomics data suggest that ABA may inhibit the SAM metabolic pathway specifically in the radicle, rather than in the hypocotyl or cotyledon. The observed inconsistencies in these experiments could potentially be attributed to species differences, though this requires further confirmation through additional studies.

Several plant hormones, including ABA, ethylene, GA, auxin, cytokinins, and brassinosteroids play an important role in regulating seed germination (Miransari and Smith, 2014). ABA negatively regulates seed germination (Nambara et al., 2010), while the others induce it (Hermann et al., 2007). It is well known that ABA, GA, and all other isoprenoids compounds such as brassinolide and zeatin are produced from IPP (isopentenyl diphosphate), which is synthesized from mevalonic acid (Nagata et al., 2003; Pawłowski, 2009; Bajguz and Piotrowska-Niczyporuk, 2023). Mevalonate kinase catalyzes the phosphorylation of mevalonic acid to (R)-5-phosphomevalonate, which can then can converted to IPP by decarboxylation and isomerization (Senbagalakshmi et al., 2019). The data from our study revealed a 2.0-fold and 2.6-fold reduction, respectively, in the relative contents of mevalonic acid and (R)-5-phosphomevalonate under ABA-treatment. This was also accompanied by a 1.3-fold decrease in the endogenous relative contents of trans-zeatin. These observations imply that ABA may restrict the biosynthesis of certain plant hormones during germination of Korean pine seed. This notion was supported by the fact that the GA and ethylene signaling pathways in Arabidopsis seeds were suppressed by ABA (Ghassemian et al., 2000; Seo et al., 2006). Similarly, it has been observed that ABA treatment decreased GA levels in *Panax notoginseng* seeds (Wang et al., 2023). It has been reported that exogenous ABA treatment increased the content of endogenous ABA (Hu et al., 2010), and promoted the expression of key genes in ABA synthesis pathway and signal transduction pathway (Hu et al., 2010; Wang et al., 2023). However, it is not clear in this study whether exogenous ABA promotes or inhibits the ABA metabolic pathway, due to the lack of measurement of the expression levels of key genes in ABA synthesis, catabolism, and signal transduction pathways. But we supposed that exogenous ABA treatment may potentially affect ABA metabolism by decreasing the levels of precursors for the synthesis of ABA.

ROS, such as H_2_O_2_, O_2_^−^, hydroxyl radicals, and superoxide radicals, are unavoidable byproducts of aerobic metabolism (Yu et al., 2016). The regulation of seed germination by ROS is concentration-dependent, with relatively low levels of ROS enhancing seed germination by promoting ABA catabolism (Choudhury et al., 2017), whereas high levels of ROS inhibit seed germination by leading to oxidative damage of cell membranes (Liu et al., 2010; Suzuki et al., 2013). The establishment of the balance between ROS production and scavenging is very important to seed germination (Wu et al., 2020b). Seeds can regulate ROS contents through metabolism of ascorbate and glutathione (Tommasi et al., 2001). In glutathione metabolism, ROS could be scavenged by the oxidation of GSH (glutathione) to GSSG (oxidized glutathione) (Hao et al., 2021). In addition, GSH could also be converted to 5-L-glutamyl-L-alanine and L-pyroglutamic acid. L-pyroglutamic acid can be further metabolized to L-glutamate, which can then be used for the regeneration of glutathione. The significant increase in the levels of these metabolites in glutathione metabolism may accelerate the GSH-GSSG cycle, which is conducive to ROS scavenging. However, in this study, the GSH amount did not significantly change, but the relative contents of GSSG increased 1.3-fold in the presence of ABA. Moreover, 5-L-glutamyl-L-alanine and L-pyroglutamic acid were found to dramatically decline. These results indicate that GSH is mainly consumed in converting to its oxidation product GSSG, rather than entering the following metabolic pathway that leads to 5-L-glutamyl-L-alanine, L-pyroglutamic acid, and ultimately L-glutamate. Thus, we supposed that the disruption of GSH-GSSG function could result in a decline in ROS scavenging ability in the radicle of ABA-treated Korean pine seeds. It is reasonable to hypothesize that the radicle of the ABA-treated seeds may be subjected to oxidative stress. These results are in line with the previous study that found that ABA inhibited *Arabidopsis thaliana* seed germination by stimulating the accumulation of ROS (Chen et al., 2022; Gong et al., 2022). Similarly, a reduction of the glutathione S-transferase associated with glutathione metabolism was observed during ABA-inhibition of germination in *Arabidopsis thaliana* (Pawłowski, 2007). In addition to glutathione metabolism, shikimic acid has been reported to have antioxidant functions, thus improving seed germination capacity (El-Soud et al., 2013; Guo et al., 2021). The relative contents of shikimate and 3-dehydroshikimic acid were decreased more than 2-fold in the radicle of ABA-treated embryos, indicating that exogenous ABA treatment disrupts antioxidative activity. In addition, chorismate, the end product of the shikimate pathway, is an important precursor for the synthesis of salicylic acid (Mishra and Baek, 2021). As a hormone, salicylic acid can promote seed germination (Lee et al., 2010a). A significant change (1.69-fold decrease in radicle and 1.92-fold decrease in hypocotyl + cotyledon) in the relative levels of salicylic acid in ABA treatment further indicate that shikimate pathway plays an important role in Korean pine seed germination.

In radicle, in addition to above-mentioned metabolism, pyrimidine metabolism, phenylalanine metabolism and galactose metabolism were also found to be influenced by ABA treatment in Korean pine seeds. However, we were unable to identify whether these three metabolic pathways are downregulated due to the detection of fewer metabolites and the varying trends observed in the presence of ABA. But these metabolic pathways are still worthy of attention for future study on ABA inhibition of tree seed germination. The involvement of pyrimidine metabolism, phenylalanine metabolism and galactose metabolism in germination inhibition induced by ABA has been reported in numerous studies (Cornelius et al., 2011; Qi et al., 2022; Wang et al., 2023).

### 4.2 The change in metabolite profiles in the hypocotyl + cotyledon during germination and their response to ABA treatment

It was found that approximately 89% of the metabolites with VIP > 1 and *P* < 0.05 in hypocotyl + cotyledon were decreased under ABA treatment. These metabolites were majorly enriched in two metabolic pathways, including glycerophospholipid and glycolysis/gluconeogenesis. Furthermore, the metabolites that are involved in these two pathways were decreased under ABA treatment, suggesting that ABA might inhibit Korean pine seed germination by repressing glycerophospholipid metabolism and glycolysis/gluconeogenesis in hypocotyl + cotyledon (**Figure 5**).

The maintenance of cell membrane stability and integrity is critical for controlling regular cell metabolism and physiological processes (Ling et al., 2022). Glycerophospholipid is an important component of the cell membrane (Calzada et al., 2016). The metabolites of the glycerophospholipid pathway, such as phosphatidic acids (i.e., a basic class of phospholipids) and phosphatidylcholine (i.e., a structural lipid with a very high content in the membrane), probably maintain the stability of cell membranes (Furse and de Kroon, 2015; Colin and Jaillais, 2020; Felczak et al., 2022). It has been reported that the down-regulation of glycerophospholipid metabolism can significantly damage the stability and permeability of the cell membrane, leading to the leakage of intracellular inclusions (Wang et al., 2019). By contrast, the level of glycerophospholipid (1-palmitoyl-sn-glycero-3-phosphocholine) was significantly increased in the root system of *Brassica napus* in response to salt stress (Wang et al., 2022). Sun et al. (2023) found that phosphatidylcholine enhanced the resistance of peach seedlings to salt stress. After Korean pine seeds were incubated with ABA for 2 weeks, we observed a remarkably strong decrease in three glycerophospholipids, including phosphatidate, phosphatidylcholine, and 1-acyl-sn-glycero-3-phosphocholine. From these data, it would appear that ABA may damage the cell membrane system in the hypocotyl + cotyledon of Korean pine seeds, presumably through the downregulation of glycerophospholipid metabolism. Relatively few investigations have attempted to characterize the role of ABA-mediated glycerophospholipid metabolism in seed germination so far. However, most studies have focused on the involvement of phosphatidic acids in ABA signal pathways, influencing the germination process (Zhang et al., 2004; Ling et al., 2022).

Seed germination is an energy-consuming process that primarily relies on glycolysis, the pentose phosphate pathway, and the tricarboxylic acid cycle for energy production, given the absence of mineral absorption systems and photosynthetic apparatus during this stage (Bonora et al., 2012; Huang et al., 2021). In the hypocotyl + cotyledon of ABA-treated seeds, the relative contents of three metabolites involved in glycolysis/gluconeogenesis, namely alpha-D-glucose, phosphoenolpyruvate, and (S)-Lactate, were significantly reduced, suggesting a suppression of the glycolysis/gluconeogenesis pathway. Until now, most researchers have demonstrated that ABA inhibits seed germination by weaking four main respiratory metabolism pathways, yet they have paid insufficient attention to the specific site of ABA’s action within the embryo. Recently, an investigation showed that ABA limited the availability of glucose in the hypocotyl region of *Arabidopsis thaliana* seed, and thus led to the inhibition of seed germination (Xue et al., 2021). Our study also uncovered an attenuated glycolysis/gluconeogenesis in the hypocotyl + cotyledon, but not in the radicle, following ABA treatment. The disparity in respiratory metabolism pathways between the hypocotyl and cotyledon merits further exploration.

## 5 Conclusion

Korean pine seeds exhibit differential metabolic processes in response to ABA treatment between the radicle and the hypocotyl + cotyledon during seed germination. Taken together, the available literature and the present results suggest that ABA treatment may inhibit Korean pine seed germination by influencing ten metabolic pathways. These metabolic pathways included pyrimidine metabolism, phenylalanine metabolism, nicotinate and nicotinamide metabolism, cysteine and methionine metabolism, phenylalanine, tyrosine and tryptophan biosynthesis, terpenoid backbone biosynthesis galactose metabolism and glutathione metabolism in the radicle, as well as glycerophospholipid metabolism and glycolysis/gluconeogenesis in the hypocotyl + cotyledon. ABA may inhibit Korean pine seed germination primarily by disrupting the biosynthesis of certain plant hormones that is mediated by cysteine and methionine metabolism and terpenoid backbone biosynthesis, as well as reducing the ROS scavenging ability, which is regulated by glutathione metabolism and the shikimate pathway in the radicle. ABA may strongly destroy the structure and function of cellular membranes by affecting glycerophospholipid metabolism, and weaken glycolysis/gluconeogenesis in the hypocotyl + cotyledon, which are also major contributors to ABA-mediated inhibition of seed germination. This study has the potential to provide fundamental insights into the metabolic mechanisms that underlie the seed germination process of Korean pine, serving as a valuable reference for understanding seed dormancy in other trees exhibiting profound physiological dormancy.

## Data availability statement

The raw data supporting the conclusions of this article will be made available by the authors, without undue reservation.

## Author contributions

YS: Funding acquisition, Methodology, Writing – original draft, Writing – review & editing. XL: Investigation, Writing – original draft. MZ: Data curation, Writing – original draft. CX: Writing – review & editing.
