# Supplementary material for: Spatial specificity of metabolism regulation of abscisic acid-imposed seed germination inhibition in Korean pine (Pinus koraiensis sieb et zucc)
Source: Front Plant Sci. 2024 Jun 20;15:1417632. doi: 10.3389/fpls.2024.1417632 (PMC11222580; doi:10.3389/fpls.2024.1417632)
Supplement: Supplementary file 1 [file Table_1.docx]

**Table S1 The relative levels of 67 metabolites in the radicle of control seeds (R) and the radicle of ABA-treated seeds (RA).**

| Metabolites | R (mean ± standard error) | | | RA (mean ± standard error) | | |
| --- | --- | --- | --- | --- | --- | --- |
| 3-Dehydroshikimic acid | 91765.2997 | ± | 25255.1919 | 26275.5659 | ± | 4494.3946 |
| 9R,10S-Epoxy-17R-hydroxy-prosta-5Z,13E-dien-1-ol methyl ester | 130908.9181 | ± | 18455.8009 | 39641.6057 | ± | 8016.1017 |
| S-Methyl-5'-thioadenosine | 108745.9655 | ± | 16873.6612 | 33979.7772 | ± | 6491.6713 |
| 3-Hydroxycapric acid | 22778.2346 | ± | 5378.2304 | 7779.4696 | ± | 2247.0261 |
| Uridine 5'-monophosphate | 814.5007 | ± | 147.6181 | 281.1083 | ± | 31.6118 |
| 1,4-Dihydroxybenzene | 16093.4068 | ± | 4288.4938 | 5750.5433 | ± | 446.9131 |
| Phenylpyruvate | 151475.1253 | ± | 19023.5368 | 58241.3707 | ± | 9008.1835 |
| (R)-mevalonic acid 5-Phosphate | 8975.7380 | ± | 1621.1166 | 3517.3253 | ± | 1536.0973 |
| N-Acetylmannosamine | 27808.4743 | ± | 5073.5383 | 13251.9870 | ± | 2015.8693 |
| Mevalonic acid | 40684.0198 | ± | 7165.4954 | 19993.0627 | ± | 3161.0764 |
| Shikimate | 11372.1973 | ± | 907.5091 | 5764.5642 | ± | 212.9762 |
| Pantothenol | 9419.0335 | ± | 1167.3337 | 4829.8428 | ± | 1112.2126 |
| Uracil | 63848.0673 | ± | 2349.0175 | 33273.7166 | ± | 4806.8244 |
| L-Gulonolactone | 112691.0398 | ± | 11978.4013 | 60164.3298 | ± | 6003.1163 |
| Eicosatrienoic Acid | 21468.8089 | ± | 2106.4018 | 11912.2347 | ± | 1592.2044 |
| L-Valine | 148895.3934 | ± | 34041.2190 | 84909.2248 | ± | 3449.5686 |
| gamma-Glutamyl-L-Methionine | 19707.3364 | ± | 756.7684 | 11332.9980 | ± | 1165.3389 |
| 5-L-Glutamyl-L-alanine | 7398.2799 | ± | 869.3387 | 4261.4836 | ± | 504.7394 |
| Salicylic acid | 64285.9112 | ± | 5464.5139 | 38093.0254 | ± | 1736.8351 |
| Lithocholic acid | 15719.2007 | ± | 1858.5291 | 9330.4520 | ± | 1542.0500 |
| L-Iditol | 1943542.5508 | ± | 120207.8476 | 1217827.8189 | ± | 136232.8304 |
| Urocanic acid | 12938.1563 | ± | 336.4964 | 8116.7074 | ± | 813.3951 |
| Alpha-D-Glucose | 60254.3793 | ± | 10309.0192 | 37963.1151 | ± | 2672.6169 |
| 3-(3-Hydroxyphenyl)propanoic acid | 42027.0756 | ± | 2811.1962 | 26666.2704 | ± | 3654.9814 |
| Dihomo-gamma-Linolenic Acid | 5180707.6554 | ± | 246552.4849 | 3296019.4600 | ± | 355224.9311 |
| L-Arabinono-1,4-lactone | 199057.7594 | ± | 8964.9581 | 127039.4233 | ± | 12428.5799 |
| Pyruvaldehyde | 35876.1066 | ± | 5205.2511 | 22932.4901 | ± | 2659.9121 |
| L-Leucine | 178489.7529 | ± | 11563.5910 | 117545.8892 | ± | 5533.9815 |
| Amygdalin | 20101.2805 | ± | 1556.8503 | 13561.7765 | ± | 821.4063 |
| 3,3',4'5-Tetrahydroxystilbene | 21705.2861 | ± | 1755.7565 | 15088.5530 | ± | 765.1805 |
| L-Alanine | 23694.3794 | ± | 2052.3603 | 16492.7434 | ± | 1919.2786 |
| N-Carboxyethyl-g-aminobutyric acid | 6951.6561 | ± | 607.2685 | 4884.6138 | ± | 264.3039 |
| Allantoin | 35111.5069 | ± | 2173.8614 | 24747.6973 | ± | 1089.4765 |
| Heptadecanoic acid | 436708.5896 | ± | 15535.8655 | 315520.3148 | ± | 25448.1769 |
| L-Isoleucine | 452012.8070 | ± | 18872.7016 | 327167.7820 | ± | 28832.9841 |
| Tyramine | 426865.5806 | ± | 18197.8219 | 316585.3124 | ± | 15455.1578 |
| trans-Zeatin | 2035.9593 | ± | 118.3865 | 1594.2226 | ± | 87.5975 |
| Azelaic acid | 10291.1038 | ± | 731.1470 | 8235.8227 | ± | 425.5112 |
| DL-2-Aminoadipic acid | 11840.4693 | ± | 484.3384 | 9631.9395 | ± | 447.3281 |
| (E)-4-Hexen-1-ol | 6774.9378 | ± | 311.9999 | 5521.9713 | ± | 164.5734 |
| Palmitic acid | 9308649.7820 | ± | 375829.5093 | 7711257.4840 | ± | 559218.3182 |
| 3-Phosphoserine | 3427.0246 | ± | 181.4185 | 2864.6153 | ± | 115.8153 |
| Glutaric acid | 12768.7160 | ± | 592.2914 | 10838.7988 | ± | 237.3257 |
| L-Pyroglutamic acid | 203121.9071 | ± | 9829.7372 | 173242.8010 | ± | 7358.7675 |
| Diethanolamine | 1110396.2624 | ± | 55614.6631 | 963755.7468 | ± | 18427.7646 |
| N-Acetyl-D-glucosamine | 491377.0258 | ± | 20266.1319 | 428163.7137 | ± | 16197.9340 |
| L-Ribulose | 89565.3345 | ± | 2894.4670 | 79442.7806 | ± | 2309.9288 |
| Trigonelline | 976560.2921 | ± | 71376.4885 | 1232425.2478 | ± | 63190.0993 |
| Guanosine | 43027.6353 | ± | 1947.2310 | 54504.8817 | ± | 3350.7455 |
| Glutathione disulfide | 219466.2972 | ± | 16475.3839 | 285686.4047 | ± | 18790.4338 |
| Pro-Ser | 7079.7408 | ± | 658.6207 | 9486.6419 | ± | 798.6248 |
| Cytidine | 94664.0389 | ± | 8673.4682 | 127898.9933 | ± | 9487.8011 |
| L-Methionine | 7483.8627 | ± | 260.7427 | 10129.7447 | ± | 800.2245 |
| 2'-Deoxy-D-ribose | 42930.0539 | ± | 2361.1988 | 65358.7949 | ± | 7365.9253 |
| Adenosine monophosphate | 4659.0740 | ± | 417.0571 | 7737.0463 | ± | 1141.7189 |
| Nicotinamide adenine dinucleotide | 2569.3217 | ± | 357.6002 | 4394.8028 | ± | 577.3069 |
| UDP-N-acetylglucosamine | 4632.4480 | ± | 328.5638 | 8126.5408 | ± | 459.9088 |
| beta-Estradiol | 1335.5395 | ± | 395.2252 | 2433.1535 | ± | 307.2114 |
| 3'-O-methyladenosine | 5748.5504 | ± | 356.7046 | 10623.4160 | ± | 1506.6156 |
| Arg-Thr | 9893.2702 | ± | 1603.6772 | 18491.9652 | ± | 3252.7173 |
| Cytidine 5'-diphosphocholine (CDP-choline) | 4785.1701 | ± | 841.6622 | 9327.1621 | ± | 479.8447 |
| L-Asparagine | 16860.2025 | ± | 1344.0752 | 33847.8014 | ± | 8915.7975 |
| Dodecanoic acid | 63618.3822 | ± | 8053.5034 | 138197.8824 | ± | 12396.5969 |
| UDP-D-Galactose | 1056.9673 | ± | 106.7670 | 2496.9139 | ± | 367.9765 |
| Ethyl glucuronide | 8750.6459 | ± | 1291.6642 | 24417.1037 | ± | 4056.7627 |
| Uridine 5'-diphosphate | 1144.2399 | ± | 119.7352 | 3362.7983 | ± | 475.3766 |
| (+)-Abscisic acid | 88553.3044 | ± | 22221.5951 | 897112.1707 | ± | 125798.6323 |
