# Supplementary material for: Spatial specificity of metabolism regulation of abscisic acid-imposed seed germination inhibition in Korean pine (Pinus koraiensis sieb et zucc)
Source: Front Plant Sci. 2024 Jun 20;15:1417632. doi: 10.3389/fpls.2024.1417632 (PMC11222580; doi:10.3389/fpls.2024.1417632)
Supplement: Supplementary file 2 [file Table_2.docx]

**Table S2 The relative levels of 62 metabolites in the hypocotyl + cotyledon of control seeds (HC) and the hypocotyl + cotyledon of ABA-treated seeds (HCA).**

| Metabolites | HC (mean ± standard error) | | | HCA (mean ± standard error) | | |
| --- | --- | --- | --- | --- | --- | --- |
| (+)-Abscisic acid | 83644.3490 | ± | 7941.7041 | 858312.7542 | ± | 137131.9652 |
| Methylmalonic acid | 26255.6264 | ± | 5410.2471 | 83179.4985 | ± | 10208.3606 |
| Dodecanoic acid | 64393.9697 | ± | 8749.1503 | 146751.7044 | ± | 12305.6702 |
| Isobutyric acid | 12408.8560 | ± | 3085.5170 | 24121.3025 | ± | 4108.2925 |
| meso-Tartaric acid | 2528.0323 | ± | 216.0714 | 3692.9237 | ± | 427.0550 |
| Myristic acid | 228190.1742 | ± | 3960.5662 | 294535.5173 | ± | 26425.4219 |
| (R)-mevalonic acid 5-Phosphate | 7076.4312 | ± | 294.2033 | 8789.7848 | ± | 700.2963 |
| Phosphoenolpyruvate | 1546.1133 | ± | 63.7800 | 1283.7790 | ± | 75.2382 |
| Adenosine | 10872322.2154 | ± | 347796.7711 | 8475921.6086 | ± | 397272.3795 |
| Diethanolamine | 1332642.9974 | ± | 61765.4155 | 1016605.7167 | ± | 57000.9730 |
| (S)-Lactate | 54831.8411 | ± | 3898.6000 | 40658.6116 | ± | 2044.5451 |
| Argininosuccinic acid | 1558298.8364 | ± | 106225.9066 | 1141273.3715 | ± | 139544.3122 |
| Citramalic acid | 45333.3529 | ± | 3519.1420 | 32942.8445 | ± | 3060.5140 |
| 4-androsten-17beta-ol-3-one glucosiduronate | 2131.7216 | ± | 130.5764 | 1504.8228 | ± | 152.6254 |
| Confertifoline | 258166.4847 | ± | 3131.9717 | 181540.8195 | ± | 20081.6904 |
| Succinate | 85456.8819 | ± | 8280.4560 | 59485.4775 | ± | 5443.8097 |
| Thymidine | 290064.0388 | ± | 24606.8939 | 201538.8496 | ± | 23318.3443 |
| 2'-O-methylguanosine | 6779.0573 | ± | 514.8394 | 4679.5229 | ± | 344.6679 |
| Arachidonic Acid (peroxide free) | 78985.5714 | ± | 2936.2687 | 54237.0584 | ± | 6064.3298 |
| L-Isoleucine | 515815.5078 | ± | 53051.8629 | 349676.7037 | ± | 34929.6500 |
| L-Ribulose | 64653.5871 | ± | 4656.4115 | 43234.3657 | ± | 4269.6968 |
| hydrocortisone acetate | 4548.9015 | ± | 200.5636 | 3000.8254 | ± | 128.5459 |
| Ribothymidine | 21089.4416 | ± | 1465.2377 | 13891.7847 | ± | 1130.4224 |
| 3,3',4,5-tetrahydroxy-trans-stilbene | 15603.6875 | ± | 1310.4331 | 10225.3057 | ± | 1437.0845 |
| Amygdalin | 6193.7292 | ± | 320.5810 | 4004.6593 | ± | 440.3768 |
| 2'-Deoxy-D-ribose | 81423.4773 | ± | 11649.6403 | 51467.7006 | ± | 4239.7054 |
| Phosphatidate | 7088.5538 | ± | 322.0806 | 4438.4733 | ± | 500.7498 |
| Pyruvaldehyde | 22624.9006 | ± | 3400.6678 | 13446.0269 | ± | 951.3266 |
| Phosphatidylcholine thioetheramide | 66372415.6280 | ± | 4093308.5078 | 39261186.6420 | ± | 10418634.6636 |
| Heptadecanoic acid | 646130.6129 | ± | 18082.7597 | 377659.0568 | ± | 69733.4976 |
| 3-(3-Hydroxyphenyl) propanoic acid | 15474.4754 | ± | 1066.4315 | 8967.1470 | ± | 1646.6226 |
| alpha-D-Glucose | 41578.5593 | ± | 2177.7274 | 24051.4580 | ± | 1919.0089 |
| 3'-O-methylguanosine | 1929.2908 | ± | 150.0402 | 1113.4522 | ± | 169.1325 |
| gamma-Glutamyl-L-Methionine | 15882.2154 | ± | 1207.9810 | 8858.6077 | ± | 985.9514 |
| Dihomo-gamma-Linolenic Acid | 6855011.5736 | ± | 366664.3095 | 3806510.5192 | ± | 697637.1552 |
| Phosphatidylcholine | 1028835.5988 | ± | 103939.4387 | 570054.3023 | ± | 164646.0858 |
| Tyramine | 555329.5540 | ± | 53562.2892 | 302761.3299 | ± | 32094.7722 |
| 9R,10S-Epoxy-17R-hydroxy-prosta-5Z,13E-dien-1-ol methyl ester | 169783.2904 | ± | 30512.8956 | 90825.7363 | ± | 8517.0963 |
| 1-Palmitoyl lysophosphatidic acid | 29705.2024 | ± | 5123.5342 | 15769.7338 | ± | 2013.4254 |
| Prostaglandin A1 | 85087.7080 | ± | 11234.1769 | 44948.7077 | ± | 2768.2494 |
| Salicylic acid | 18798.6600 | ± | 2493.1065 | 9810.4866 | ± | 1114.1968 |
| 1-Acyl-sn-glycero-3-phosphocholine | 2762277.2352 | ± | 351750.8272 | 1441180.9789 | ± | 258837.5948 |
| Pristanic acid | 43337.6235 | ± | 3780.5611 | 22248.2757 | ± | 5626.8982 |
| Heneicosanoic acid | 27567.9870 | ± | 2656.8282 | 14092.4026 | ± | 3102.5467 |
| 2'-Deoxyuridine | 31245.3860 | ± | 3565.3896 | 15027.1647 | ± | 1530.5633 |
| 2-hydroxy-butanoic acid | 13763.7824 | ± | 1314.3114 | 6499.0394 | ± | 922.5831 |
| D-Arabinonic acid, gamma-lactone | 666568.4307 | ± | 78957.3550 | 303395.4832 | ± | 32236.3828 |
| L-iditol | 720185.6284 | ± | 123750.6219 | 324547.8515 | ± | 38375.4285 |
| Arachidic acid | 6214.1336 | ± | 954.0632 | 2798.2890 | ± | 1027.3761 |
| Eicosatrienoic Acid | 31697.2678 | ± | 4596.4598 | 13698.6564 | ± | 4010.9817 |
| Behenic acid | 127793.7283 | ± | 8718.6723 | 54414.2069 | ± | 15727.3482 |
| Tricosanoic acid | 39981.6663 | ± | 2926.8174 | 16386.9417 | ± | 4642.9862 |
| Tetracosanoic acid | 60153.2330 | ± | 4491.1327 | 23589.7824 | ± | 6998.7863 |
| Oleanolic acid | 18124.4128 | ± | 1287.6615 | 6976.6768 | ± | 1753.6868 |
| Eicosadienoic Acid | 201172.7962 | ± | 9287.1004 | 74701.7815 | ± | 21434.9550 |
| Hesperetin | 4069.0399 | ± | 620.6072 | 1378.7316 | ± | 347.4975 |
| Eicosapentaenoic acid | 1883720.9524 | ± | 178248.6044 | 617722.3273 | ± | 123851.9713 |
| 15-Keto-prostaglandin E1 | 126587.7482 | ± | 25823.2112 | 40890.1859 | ± | 5759.0664 |
| 1,4-Dihydroxybenzene | 7278.2200 | ± | 2627.1550 | 2223.3174 | ± | 241.1074 |
| Threonic acid | 188380.7948 | ± | 19916.7803 | 55334.4554 | ± | 15782.9552 |
| 3-Dehydroshikimic acid | 39648.8617 | ± | 15027.0141 | 8322.0607 | ± | 1178.4092 |
| Cholesteryl sulfate | 88461.9975 | ± | 16449.0106 | 15113.7271 | ± | 6075.6934 |
